# HIV incidence and adherence after pre-exposure prophylaxis initiation in key populations in Indonesia: Findings from a real-world pilot program 2021-2023

**DOI:** 10.1016/j.ijregi.2025.100573

**Published:** 2025-01-19

**Authors:** Nadia Hanum, Miasari Handayani, Armina Padmasawitri, Zulfan Zazuli, Kusnandar Anggadiredja, Mawar N. Pohan, Tarinanda A. Putri, Fani F. Rakhmat, Dwi S. Anggiani, Nurhalina Afriana, Endang Lukitosari, Bagus R. Prabowo, Rudi Wisaksana

**Affiliations:** 1 Department of Pharmacology and Clinical Pharmacy, School of Pharmacy – Bandung Institute of Technology, Bandung, Indonesia; 2 Biosciences and Biotechnology Research Center – Bandung Institute of Technology, Bandung, Indonesia; 3 Research Center for Care and Control of Infectious Disease – Padjajaran University, Bandung, Indonesia; 4 HIV and STI Working Group of the Directorate General for Disease Prevention and Control – Indonesian Ministry of Health, Jakarta, Indonesia; 5 The Joint United Nations Programme on HIV/AIDS (UNAIDS), Jakarta, Indonesia

**Keywords:** Pre-exposure prophylaxis, HIV prevention, Key populations, Indonesia, Real-world study

## Abstract

**Objectives:**

To provide important data for the national rollout, Indonesia launched a pre-exposure prophylaxis (PrEP) pilot program. In this study, we determined the HIV incidence and adherence among key populations, PrEP initiation, sexually transmitted infection incidence, and changes in sexual behaviors.

**Methods:**

The Indonesian PrEP pilot program (December 2021 to December 2023, rolled out in 21 districts in 10 provinces) was a longitudinal, non-randomized implementation study among men who have sex with men, female sex workers, transgender women, people who inject drugs, and serodiscordant partners of people with HIV. Eligible participants were offered same-day oral PrEP and completed the baseline, 1-monthly, and 3-monthly follow-up visits.

**Results:**

A total of 9124 individuals initiated PrEP between December 2021 and December 2023; 4220 had at least one follow-up visit. Of the 4220, 31 seroconverted over 2817.5 person-years; overall incidence rate (IR) 1.10 (95% confidence interval 0.75-1.56) per 100 person-years. All seroconversions occurred during poor adherence or PrEP discontinuation (IR 7.8; 5.34-11.16 vs 0; 0-0.4 for adequate adherence). Event-driven PrEP users showed poorer adherence than daily PrEP users (*P <*0.001). In men who have sex with men, HIV incidence was higher in those who lived outside Java and Bali (adjusted IR ratio 5.56; 1.68-18.38, *P* = 0.005).

**Conclusion:**

Insufficient adherence leads to decreased efficacy of PrEP. Improving adherence and access to PrEP is a public health priority in Indonesia.

## Highlights

• This study provides the first estimates of HIV pre-exposure prophylaxis (PrEP) efficacy in Indonesia.
• Key populations faced challenges in event-driven and daily PrEP taking.
• All HIV seroconversions were related to non-adherence or having discontinued PrEP.
• There were unmet PrEP criteria among people living in areas with limited PrEP services.
• Strategies to strengthen PrEP adherence and PrEP delivery to all are urgently needed.

## Graphical abstract

**Figure:**
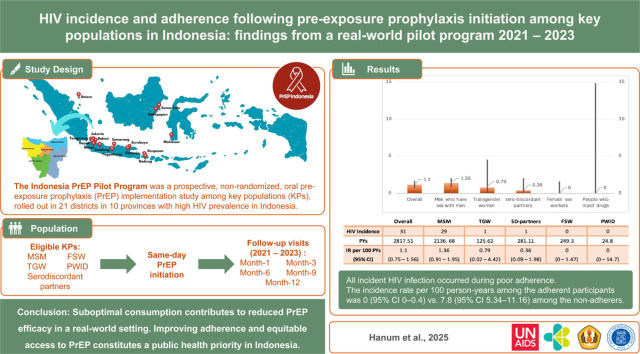

## Introduction

Indonesia continues to rank among the countries in Asia and the Pacific with the highest rate of new HIV infections [1]. As of 2023, an estimated 546,000 people are living with HIV, with a prevalence of 0.34% across all age groups and 25,740 new infections [2]. The HIV incidence per 1000 population in 2022 was 0.09 (0.08-0.10), whereas the HIV prevalence rate in the adult general population was 0.3 (0.3-0.4) [3,4]. Although the number of new HIV infections has decreased by 52% since 2010, nearly half of all new HIV infections occur in young people [1,4]. In most provinces, Indonesia's HIV epidemic is concentrated among key populations (KPs), including men who have sex with men (MSMs), female sex workers (FSWs), people who inject drugs (PWIDs), and transgender women (TGWs), except for five provinces of Tanah Papua, which have a generalized epidemic with an estimated HIV prevalence of 2.3% in 2023 [2]. The median HIV prevalence in KPs in 2022 remains much higher than the general adult population: 17.9% in MSMs, 13.7% in PWIDs, 11.9% in transgender people, and 2.1% in FSWs [1].

Clinical trials and observational cohort studies have shown that pre-exposure prophylaxis (PrEP) is highly efficacious in preventing HIV [[5], [6], [7]]. However, global implementation of PrEP has been slow, and there is a paucity of data from low- and middle-income countries (LMICs) examining the uptake and real-world efficacy of PrEP to guide implementation [[8], [9], [10]]. To date, most data have been generated from studies conducted in Europe, North America, and Australia [[11], [12], [13]]. Despite the continued expansion of the PrEP strategy, there are sociocultural contexts and health care infrastructure gaps between LMICs (including Indonesia) and high-income countries, which present particular challenges for the former in PrEP implementation, necessitating a cautious approach [14]. Real-world experiences in diverse populations and settings are necessary.

Indonesia decided to pilot a PrEP program in 2021-2023 in provinces with high levels of HIV prevalence to reduce new HIV infections and test the feasibility of large-scale national implementation. Specifically, the program aimed to collect empirical data to determine effective ways to implement unrestricted PrEP in Indonesia because high-quality evidence supporting the feasibility of implementation (i.e. efficacy, adherence, retention, and risk compensation) is lacking [15]. In this study, we determined HIV incidence, PrEP adherence, PrEP initiation, incidence of sexually transmitted infections (STIs), and changes in sexual behaviors in the Indonesia PrEP Pilot Program.

## Methods

### Study design and participants

The Indonesian PrEP pilot program was a longitudinal, nonrandomized implementation study of oral daily (D) or event-driven (ED) tenofovir disoproxil fumarate and emtricitabine as PrEP in HIV-negative KPs at an increased risk of HIV infection in Indonesia [16,17]. It involved 60 health care facilities (hospitals, private clinics, and government clinics) spread across 21 districts in 10 provinces with a high HIV prevalence (Table 1 in Supplemental File-1), that were designated by the Indonesian Ministry of Health as ready to deliver PrEP services. We summarize the study protocol in Supplemental File-1, whereas the detailed program guidelines are available online [18].

The participants were KPs belonging to one of the four populations: MSMs, FSWs, PWIDs, and TGWs. In addition, we recruited serodiscordant partners from individuals infected with HIV. Eligibility criteria included those who were HIV-negative aged 17 years and older; at substantial risk of HIV transmission by reporting at least one of the following sexual behaviors in the past 3 months: more than one sexual partner, inconsistent condom use, condomless anal sex, STIs history, ever used PrEP or postexposure prophylaxis (PEP), and having an partner who is HIV-positive who was not using antiretrovirals, or using antiretrovirals irregularly in the past 6 months, or without known viral loads (VLs), or with unsuppressed VLs (>1000 copies/ml) after 6 months of treatment, or planning to have children with unsuppressed VLs; and had no indications of having acute HIV infection and no contraindications to the regimen.

### PrEP Indonesia model

PrEP delivery was designed as a facility- and community-based model (Table 2 in Supplemental File-1), whereby the PrEP program was provided by health care facilities with the involvement of HIV and KP communities [19].

#### Pre-enrollment

Peer educators or outreach workers were involved greatly in recruiting participants through HIV communities, sex work venues, clinic-based referrals, word of mouth, and social media campaigns. Voluntary counseling and testing clients and serodiscordant couples attending health care facilities were also recruited. To assess sexual behavior, prospective users were asked to complete an online (via a link to a web page) or paper questionnaire independently (in Indonesian), with peer educators in communities or with HIV counselors in facilities, regarding their recent sexual behaviors. Medical screening was performed at the HIV Care, Support and Treatment (CST) services for eligible individuals and included tests for HIV, STIs, hepatitis B and C, and creatinine levels. The participants were counseled about the program procedure before initiation.

#### Study procedures

Eligible participants were offered D-PrEP on the same day or within 1 week of PrEP initiation; MSMs could also opt for ED-PrEP. The choice of regimen was decided after a clinical risk assessment and discussion between the prescribing clinician and the participant. PrEP initiation began on December 1, 2021 and the follow-up ended on December 31, 2023. Participants completed baseline and follow-up visits at 1 month and then every 3 months (months 3, 6, 9, 12, and so on).

For each clinic visit, the following procedures were performed: HIV and STI testing following the national guidelines [20], assessment of side effects and adverse events, adherence counseling, and return of the leftover tenofovir disoproxil fumarate and emtricitabine to the clinic in exchange for a refill to cover their needs until the next clinic visit (maximum of 90 tablets for 3 months). Participants were also asked about their past 3 months’ number of sexual partners, consistency in condom use, condom use during last sex, and past week's sex frequency. Besides the 3-monthly follow-up visits, non-routine visits could also be made if there were side effects of STIs, risk reduction and adherence counseling, other clinical tests, and for ED-PrEP users to take medication. PrEP needs were reassessed at each visit; a new prescription was dispensed if required and participants could switch regimens if appropriate. A PrEP mobile application was used to provide information on how to take PrEP and book clinic visits as a calendar to mark their sexual activities and PrEP-taking patterns and to remind them of upcoming scheduled visits (Subsection 2.4, Supplemental File-1).

If participants seroconverted to HIV, their participation was terminated and referrals were provided to HIV services. Participants could also terminate their participation for the following reasons: experiencing side effects or adverse drug reactions, problems with compliance, or feeling no longer at risk due to changes in behavior or lifestyle.

### Measures and outcomes

The data captured in this study included age or date/year of birth, sex, KPs, area of residence, healthcare facilities, sexual behaviors (number of male and female sex partners, consistent condom use, sex frequency in the past week, and condom use at last sex), PrEP prescriptions (number of tablets dispensed), HIV and STI test results, adherence score, PrEP use status (active, discontinued, switching regimen, and seroconversion), side effects, and other clinical evaluation results.

The main efficacy outcomes were incidence of HIV infection among all KPs and adherence to PrEP. Secondary outcomes were PrEP initiation, PrEP continuation, STI incidence, and changes in sexual behavior during follow-up. Outcomes definitions and how they were assessed are shown in Table 1. The adherence rate was measured as the percentage of days an individual was covered by PrEP, equal to the total number of actual drugs used divided by the number of times the drugs should have been taken during the follow-up period. For ED-PrEP users, we evaluated PrEP protection per day, during which sexual activity data were reported to counselors or recorded on the mobile app. Correct ED-PrEP use was defined as two pills 2-24 hours before a sex act, followed by one tablet every 24 hours after the last sex act [21]. Adherence scores of 95% were defined as adequate (Table 1).

**Table 1:** Outcome measures and the definitions.

Outcome measures	Definitions and assessment
HIV Incidence	Seroconversion from HIV-negative status at enrolment to HIV-positive status during follow-up per 100 PYs in participants with at least one follow-up visit, stratified by key populations; Assessed through laboratory records.
PrEP adherence	Defined as all participants with at least one follow-up visit: • Adequate adherence: adherence score of 95% and above; • Poorer adherence: adherence score between 40% and 94% • Very poor adherence: adherence score of below than 40%, including non-adherence or not using PrEP due to lost to follow-up or late visit; Informed by dispensing and return records of unused tablet count combined with clinicians/HIV counsellors/peer educators’ assessment based on participants’ self-report adherence and sexual behaviors.
PrEP initiation	Received PrEP on the day or within a week of study enrolment among all eligible for PrEP; Assessed through CST service and pharmacy dispensing records.
PrEP continuation	Received a PrEP drug refill at 1, 3, 6, and 9 months consecutively in those who initiated PrEP the latest 9 months before 31 December 2023. PrEP users who initiated PrEP for a shorter time than the specified period before the data collection end date were excluded due to the insufficient follow-up time to measure continuation; Assessed through CST service and pharmacy dispensing records.
STI Incidence	Any STI diagnosis per 100 PYs in those with at least one follow-up visit with no report of STI at baseline screening; Assessed through laboratory records.
Changes in sexual behavior measures	Changes over follow-up visit in consistent condom use in the past 3 months (always or mostly use PrEP during sexual intercourse), condom use at last sex, and multiple sex partners (two or more sexual partners) in the past 3 months in participants with at least one follow-up visit, from the first until last follow-up visit; Assessed through self-reported sexual behavior questionnaire at each follow-up visit.

PrEP, pre-exposure prophylaxis; PY, person-years; STI, sexually transmitted infection.

### Statistical analysis

Descriptive statistics were used to summarize the characteristics of the KPs enrolled in the study and assess PrEP initiation. Trends in sexual behaviors, adherence, and STI diagnoses during follow-up were analyzed using univariate generalized estimating equation models with a logit link, accounting for multiple responses from individuals.

For the analysis of HIV incidence, person-years (PYs) of follow-up were calculated from the date of PrEP initiation until (i) the date of incident HIV infection for individuals who seroconverted, (ii) the date of the last follow-up visit with an HIV-negative test before the censoring date (December 31, 2023) for participants who did not seroconvert, or (iii) the last date of visit for those who discontinued PrEP or were lost to follow-up (missed three consecutive follow-ups). We adopted a single random-point method by sampling infection dates from a uniform distribution to impute HIV infection dates between the first HIV-positive and last HIV-negative test results [22]. For any STI incidence, we used the date of the first positive STI test and PYs extended to the last STI test.

HIV and STI incidence rates (IRs) were reported with 95% confidence intervals (CIs) calculated using probabilities from the exact Poisson method. Determinants of HIV incidence were assessed using mixed-effect Poisson regression models (two-level random-intercept models with regions defining the second level, considering clustering according to PrEP sites). IR ratios adjusted for age and adherence status with their 95% CIs were presented. Missing data were excluded from the analysis. All analyses were performed using the Stata 15 software (StataCorp LLC, Texas, USA).

## Results

Between December 2021 and December 2023, 17,584 individuals who were HIV-negative were screened for PrEP eligibility, of whom 16,469 (93.67%) were found to have an increased risk of HIV infection, and 9124 (55.40% of those eligible) initiated PrEP. Of the 9124, 4220 participants underwent at least one follow-up visit (Figure 1 in Supplemental File-2). PrEP initiators were older than those who did not, eligible women were more likely than men to initiate PrEP, as were FSWs and TGWs, than MSMs and serodiscordant partners of people living with HIV (Table 2). Eligible individuals living in Java and Bali (more developed provinces) were more likely to initiate PrEP than those living in other provinces. Although eligible, 629 individuals living in provinces not covered by the program failed to initiate PrEP. Those who initiated PrEP reported a higher number of male sex partners and a higher sex frequency. No differences were observed in STI diagnoses.

**Table 2:** Baseline characteristics individuals eligible for PrEP.

Characteristics	Eligible and initiated PrEP (N = 9124)		Eligible but did not start PrEP (N = 7345)		*P*-value^a^
	n	%	n	%	
**Demographics**					
**Age (years)**	8988 obs		6544 obs		**<0.001**
median (IQR)	28	(24-33)	27	(23-31)	
mean (SD)	29.80	(16.26)	28.67	(37.67)	
<20	298	3.32	256	3.91	
20-24	2,368	26.35	2,066	31.57	
25-49	6,118	68.07	4,153	63.46	
50+	204	2.27	69	1.05	
**Sex**	9124 obs		7345 obs		**<0.001**
Male	7758	85.03	6701	91.23	
Female	1366	14.97	644	8.77	
**Key populations**	9124 obs		7345 obs		**<0.001**
MSM	6790	74.42	5704	77.66	
FSW	1117	12.24	414	5.64	
PWID	21	0.23	35	0.48	
TGW	482	5.29	171	2.33	
Sero-discordant partners	712	7.80	1,021	13.90	
**Provinces**	9124 obs		7275 obs		**<0.001**
Java and Bali^b^	8391	91.97	6130	84.26	
Outside Java and Bali^b^	729	7.99	516	7.09	
Other provinces^b^	4	0.04	629	8.65	
Bali	1277	14.00	382	5.20	
Banten	454	4.98	504	6.86	
DI Yogyakarta	363	3.98	276	3.76	
DKI Jakarta	3624	39.72	2289	31.16	
West Java	1273	13.95	1644	22.38	
Central Java	446	4.89	393	5.35	
East Java	954	10.46	642	8.74	
East Kalimantan	308	3.38	173	2.36	
Riau	86	0.94	142	1.93	
South Sulawesi	335	3.67	201	2.74	
Other provinces^b^	4	0.04	629	8.56	
**Sexual behaviors (in the past 3 months unless stated otherwise, all self-report)**					
**Number of male sexual partners**	9124 obs		7345 obs		**<0.001**
Median (IQR)	3	2-4	2	1-3	
Mean (SD)	3.29	4.77	2.58	3.43	
0-1	2165	23.73	2675	36.42	
2-4	5886	64.51	3952	53.81	
5-9	630	6.90	464	6.32	
10 or more	443	4.86	254	3.46	
**Number of female sexual partners**	7393 obs		5806 obs		0.470
Median (IQR)	0	0-1	0	0-1	
Mean (SD)	0.73	2.20	0.74	2.23	
0-1	6018	81.40	4729	81.45	
2-4	1329	17.98	1029	17.72	
5-9	31	0.42	29	0.50	
10 or more	15	0.20	19	0.33	
**Consistent condom sex**	9115 obs		7340 obs		0.175
Yes	3133	34.37	2449	33.37	
No/unknown	5982	65.63	4891	66.63	
**Condom at last sex**	9124 obs		7345 obs		0.073
Yes	4	0.04	0	0	
No	9124	99.96	7345	100	
**Sex frequency in the past week (times)**	9124 obs		7345 obs		**0.002**
Median (IQR)	0	0-0	0	0-0	
Mean (SD)	0.05	0.12	0	0	
0-1	9112	99.87	7345	100	
2-4	12	0.13	0	0	
**STI diagnoses**	9108 obs		7322 obs		0.142
Yes	851	9.34	734	10.02	
No/unknown	8257	90.66	6588	89.98	
**PrEP**					
**First-timer users**	9124 obs				
PrEP-naïve	8850	97.00			
Former users	274	3.00			
**Regimen**	9124 obs				
Daily	5954	65.25			
Event-driven	3170	34.75			
**Chosen health care facilities**	9124 obs				
Private or government clinics	8667	94.99			
Hospitals (inc. specialists)	457	5.01			

FSW, female sex workers; IQR, interquartile range; MSM, men who have sex with men; obs, obsefvations; PrEP, pre-exposure prophylaxis; PWID, people who inject drugs; TGW, transgender women.

a *P*-value from chi-square tests for categorical data.

b Java and Bali provinces are Bali, Banten, DI Yogyakarta, DKI Jakarta, West Java, Central Java, and East Java; Outside Java and Bali provinces are East Kalimantan, Riau, and South Sulawesi; Other provinces include: Aceh, Bangka Belitung, Lampung, Bengkulu, Jambi, Sumatera Selatan, Sumatera Barat, Sumatera Utara, Kalimantan Selatan, Kalimantan Barat, Kalimantan Utara, Kalimantan Tengah, Maluku, Nusa Tenggara Barat, Nusa Tenggara Timur, Papua dan Papua Barat, Sulawesi Utara, Sulawesi Barat, Sulawesi Tengah, Sulawesi Tenggara.

Of those who initiated PrEP, 97% were PrEP-naïve, 95% accessed PrEP care provided at private or government clinics, 65.2% chose D-PrEP over ED-PrEP (34.7%), and almost 92% lived in the Java and Bali provinces. The mean age was 29.8 years (SD 16.26); 85.0% were men; and 74.7% were MSMs, followed by FSWs (12.2%), serodiscordant partners (7.8%), TGW (5.2%), and PWIDs (0.2%). There were very few missing data for PrEP initiators.

### PrEP initiation

PrEP initiation increased from 57 participants in the first quarter of 2022 (including December 2021) to 1671 in the fourth quarter of 2023 (*P-*value for linear trend *<0.001*) (Figure 2 in Supplemental File-2).

### PrEP adherence and sexual behaviors

**Figure 1:**
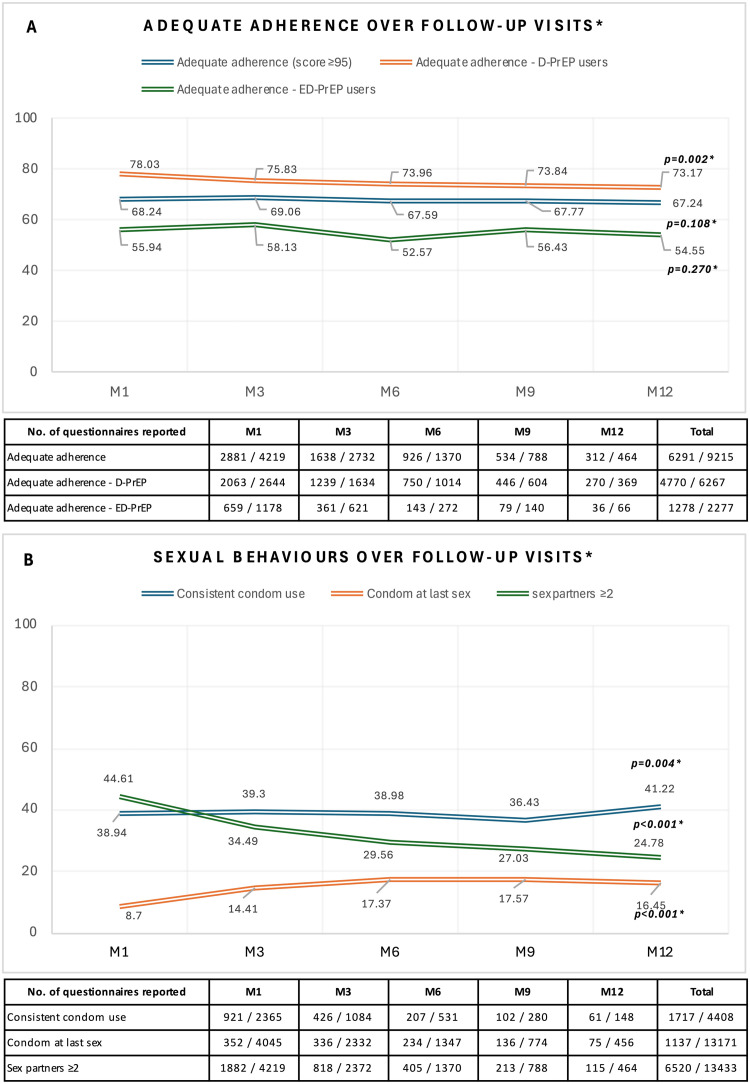
Changes in (a) adherence and (b) sexual behaviors from first to last follow-up among participants with at least one follow-up visit. *P-value for linear trend from generalized estimating equation-logistic models.

Adherence was assessed during 9215 follow-up visits of the 4220 participants with at least one follow-up visit. Adequate adherence was reported in 68.7% (6291) of the assessments, whereas 31.7% (2924) showed adherence below 95%. D-PrEP users showed better adherence (76.1% of the total assessments) than ED-PrEP users (56.1%) (*P* <0.001). Figure 1(a) shows that there were no changes in overall adherence over time (*P* = 0.108) and adherence in ED-PrEP users (*P* = 0.270), whereas D-PrEP users showed a decreasing trend (from 78% in the first follow-up to 73.1% after 12 months, *P* = 0.002). Figure 1(b) shows sexual behaviors related to HIV risk over time. Consistent condom use (*P* = 0.004) and condom use during the last sex session (*P* <0.001) increased, whereas multiple sex partners decreased (*P* <0.001).

### HIV incidence

**Figure 2:**
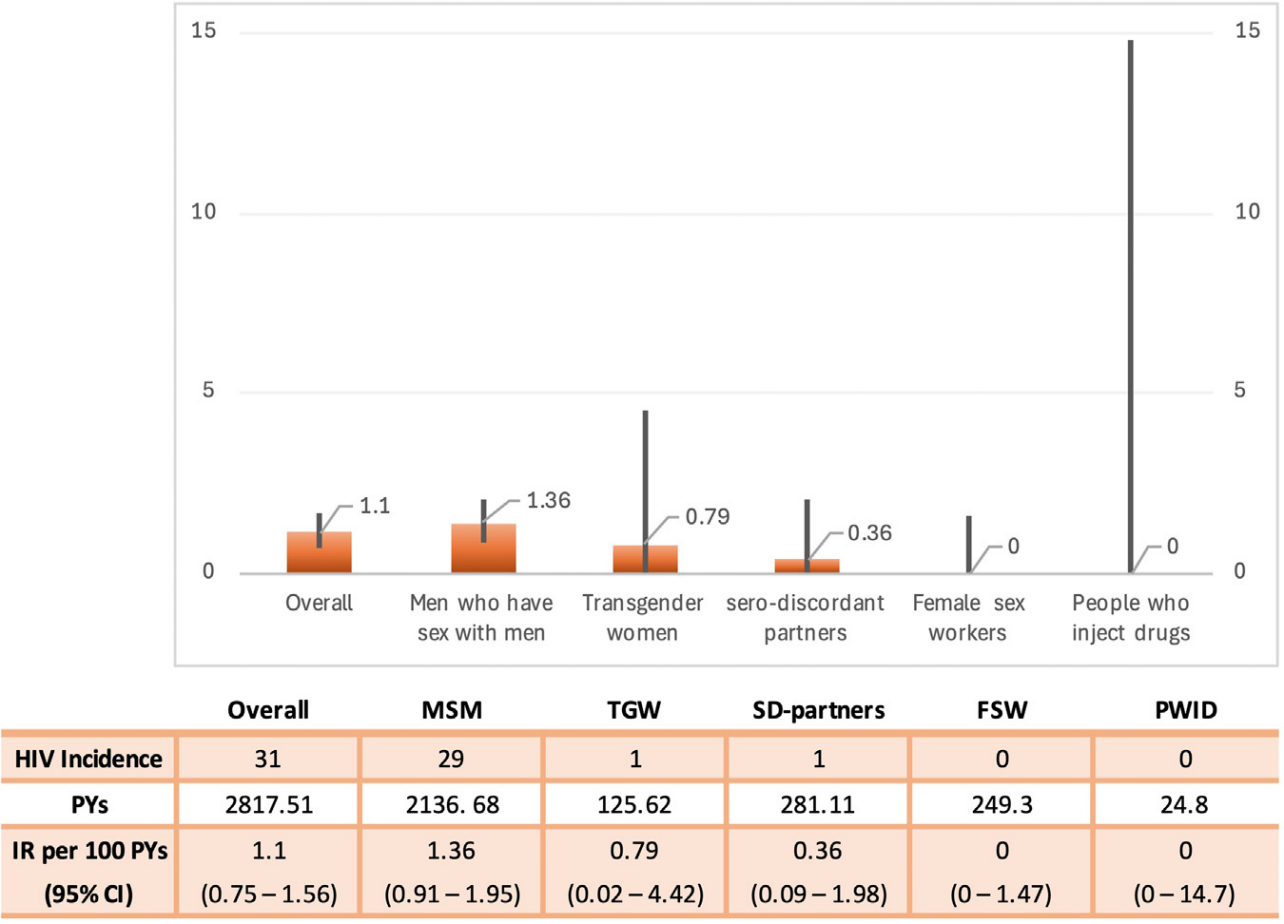
HIV incidence among key populations at increased risk of HIV infection. CI, confidence interval; FSW, female sex workers; MSM, men who have sex with men; PWID, people who inject drugs; PYs, person-years; SD, serodiscordant; TGW, transgender women.

In total, 31 of the 4220 individuals were seroconverted. With total PYs of follow-up of 2817.5, the overall incidence rate was 1.11 (0.75-1.57) (Figure 2). A total of 29 of 31 individuals who seroconverted were MSMs (IR 1.36; 0.91-1.95). No HIV seroconversion has been reported in FSWs or PWIDs.

**Figure 3:**
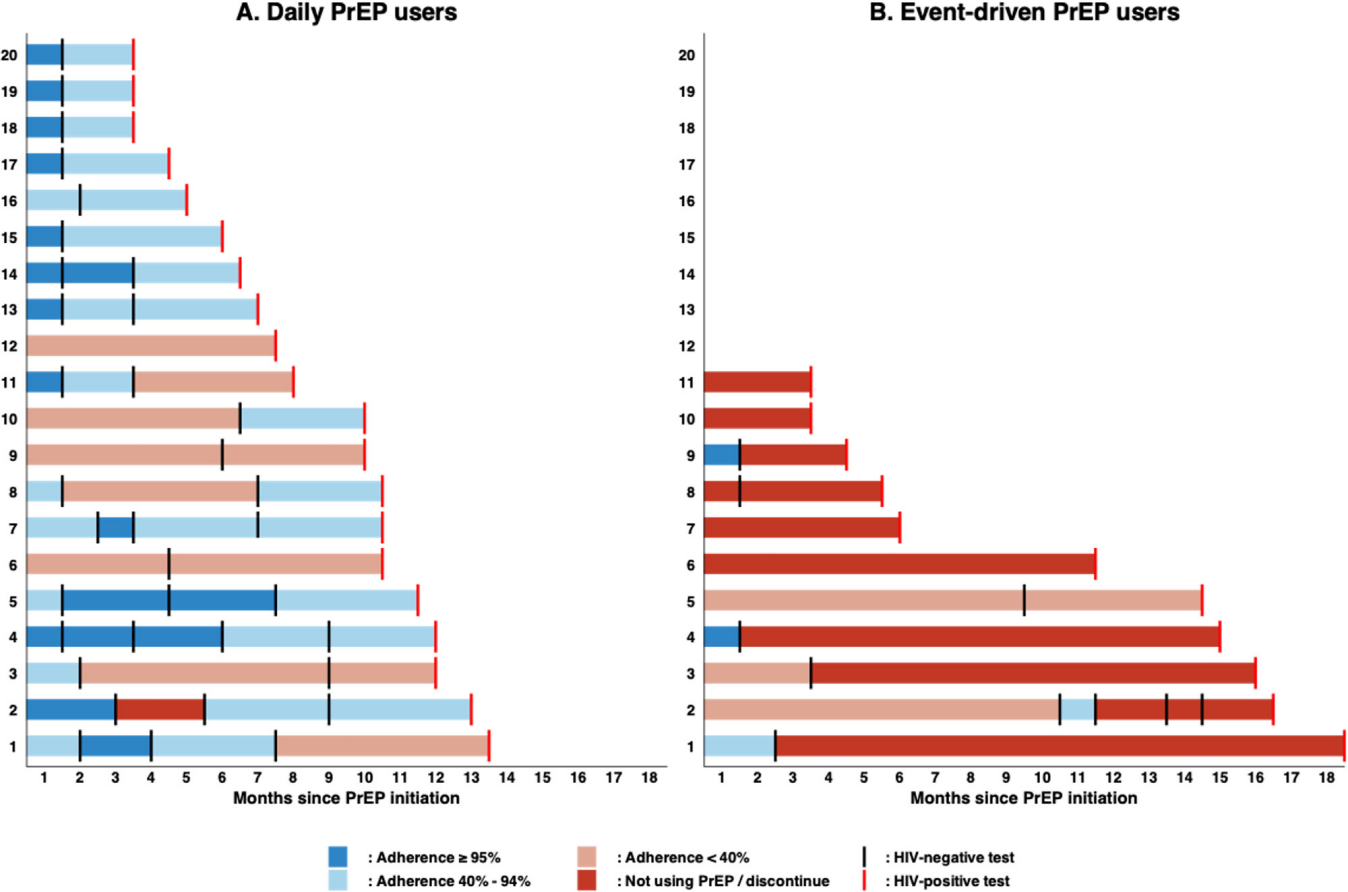
Characterization of the (a) 20 HIV seroconversions in daily-PrEP users and (b) 11 HIV seroconversions in event-driven PrEP users. PrEP, pre-exposure prophylaxis.

There were 20 seroconversions in D-PrEP users and 11 seroconversions in ED-PrEP users; all never switched their regimens (Figure 3). Of the participants who underwent HIV seroconversion who were receiving D-PrEP, six had sex acts with poor adherence (between 14% and 34%) and 14 had suboptimal adherence (between 67% and 90%) (Figure 3a). Of the ED-PrEP users, 10 acquired HIV during the period of not using PrEP or discontinuation, with six reporting condomless sex and one acquired HIV during the period of very poor adherence (<10%) according to the prescription between the last HIV-negative visit and the seroconversion visit, which was not sufficient to cover their sex acts (Figure 3b). Four participants had long durations between their last negative and first positive HIV test result (>300 days).

In MSMs, HIV incidence was higher in those living outside Java and Bali (adjusted IR ratio 5.56; 1.68-18.38, *P* = 0.005) (Table 1 in Supplemental File-2). After adjusting for age and adherence status, HIV incidence did not differ between the PrEP regimens. There were no associations between age, recent sexual behaviors, previous STI diagnosis, and subsequent HIV incidence.

### Sexually transmitted infection incidence

This analysis was performed on 3787 participants with at least one follow-up visit and without reported STI diagnoses at the baseline. There were 715 new STI diagnoses during follow-up, and the overall STI incidence was high (IR 28.89; 27.11-30.72) (Table 2 in Supplemental File-2). MSMs had the highest incidence (633 new STIs; IR 35.45; 33.28-37.67). The prevalence of positive STI diagnoses increased over time (*P* <0.001) (Figure 3 in Supplemental File-2).

## Discussion

In this first oral PrEP program in Indonesia, we documented a moderately high incidence of HIV infection in KPs because of insufficient adherence. Despite an overall incidence rate of 1.1 per 100 PYs (0.75-1.56), the 31 incident HIV infection all occurred in the context of discontinued PrEP or during the time of poor adherence, translating to an IR of 0 per 100 PYs (0-0.4) in the adherent participants.

A previous cohort study in MSMs and TGWs enrolled in private/non-government clinics in Jakarta and Bali (N = 2341) between 2017 and 2020 (before PrEP was available in Indonesia) reported incredibly high IRs per 100 PYs: 9.39 (7.78-11.17) in Jakarta and 7.24 (5.73-9.13) in Bali [23]. The IRs per 100 PYs in the subset analysis of KPs in the same regions in this study (n = 2291 MSM and TGW in Jakarta and Bali) were far lower: 1.69 (1.05-2.56) in Jakarta and 0.65 (0.07-2.36) in Bali (Table 3, Supplemental File-2), which equate to a ~82% reduction in HIV incidence in Jakarta and ~91% reduction in Bali. However, the extent to which this reduction could be because of the impact of PrEP or differences in population characteristics remains unclear.

In this study, adequate adherence was reported in 69% of the total assessments, which is in line with a recent meta-analysis that estimated 35.8% inadequate adherence worldwide and 31.5% PrEP discontinuation in MSMs and TGWs [24]. Our results imply that when considering a diverse population, suboptimal PrEP consumption and treatment discontinuations are common. This likely explains why the efficacy of PrEP in this study was lower than that reported in clinical trials and open-label studies. Low adherence to PrEP and treatment discontinuation have been found to be significant determinants of PrEP failure in previous implementation studies conducted in high- and low-income countries [25,26].

Consistent with previous studies on LMICs, ED-PrEP users reported significantly lower adherence than D-PrEP users [8,10]. Although the ED-PrEP regimen is more complicated than its daily equivalent, it is imperative to administer ED- and D-PrEPs. If there were no ED-PrEP alternatives available, a higher proportion of individuals would probably not begin PrEP and would still be at a high risk of contracting and spreading HIV. However, our findings indicate that PrEP users in Indonesia should be better informed about the adherence challenges with the former regimen, such as difficulties in scheduling sex, remembering to take the pills in the 2 days after a sex act, and the risk of HIV infection if they do not adhere to it. The present mobile PrEP app can be enhanced by including more regular and personalized reminders, such as daily or weekly inquiries on sexual activity as indicators of HIV risk. This could encourage participants to adhere to the study as a means of reducing their risk.

PWID were underrepresented in this study, and the majority of those who started PrEP were MSMs. PWIDs might be more difficult to reach out to or oral PrEP might just be an inappropriate preventive measure, whereas MSMs might have more awareness of HIV prevention. These issues must be addressed through locally relevant initiatives. To maximize the efficacy of a national PrEP program, a significant expansion of PrEP access above the level offered during the pilot period or the use of alternative PrEP methods, such as long-acting injectable options, is necessary. Within a clinical setting, the health care provider's role in evaluating and discussing PrEP needs is crucial for increasing awareness, uptake, and coverage in the most marginalized and vulnerable populations. In addition, the community's role in promoting and helping adherence must be strengthened. Although Indonesia adopted the combination of facility- and community-led PrEP models to achieve the target of PrEP services and address stigma, the fact that only 55% of eligible participants started PrEP emphasizes how vital it is to guarantee equitable access to PrEP for everyone.

PrEP initiation also varies by region. In addition to being less likely to begin PrEP, residents of provinces outside Java and Bali, which, incidentally, had less qualified and designated healthcare facilities, were also at a higher risk of HIV incidence. Access can also be hampered by stigma surrounding HIV and PrEP, which is pervasive in many areas of Indonesia. This is an unmet PrEP need that must be addressed by expanding the number of PrEP providers and providing assistance for peer guiding and community signposting in those provinces.

We found a higher level of PrEP initiation in those who reported higher risk of sexual behaviors. A previous cross-sectional survey in Bali reported a high level of interest in PrEP use among those who perceived themselves to be at risk [15]. This is encouraging because we also did not observe changes in behavioral disinhibition. However, participants had high rates of STI diagnoses, comparable to the results reported in other PrEP implementations in LMICs [27] but lower than those found in PrEP cohorts from high-income countries [28]. Regarding each bacterial STIs, distinct regional patterns have been documented, although some discrepancies in estimates maybe explained by variances in methodology. Doxycycline PEP, which is currently being adopted in a few countries [29], has been demonstrated to lower the risk of contracting bacterial STIs [30]. Although doxycycline PEP is not yet available in Indonesia, it may be taken into consideration in the future for specific PrEP users, for example, those with recurrent STI diagnoses. In addition, tailored interventions such as thorough sexual behavior counseling, partner notification approaches, self- or home-based testing, and bacterial STI vaccinations might be effective in reducing STI transmission.

This study has several limitations that should be considered when interpreting the results. Adherence and risky sexual behaviors were self-reported or relied on assessments by health care workers. Participants may have underestimated such behaviors because of social desirability, which could have overestimated actual adherence. Nonetheless, we did not find any seroconversion in those who adhered to PrEP, confirming self-reported adherence. Owing to logistical and financial constraints, not all health care facilities have the necessary resources to quantify the plasma concentrations of PrEP drugs and perform genetic resistance testing in all seroconverted participants. Therefore, we were unable to verify the participants’ self-reported adherence by examining their PrEP consumption. We did not perform regular laboratory screening for specific types of bacterial STIs (gonorrhea, chlamydia, or syphilis) in all health care facilities; therefore, we were unable to demonstrate the impact of PrEP use on specific types of bacterial STIs. Finally, data on important sociodemographic characteristics, such as education level, employment, and lifestyle, were not collected in this pilot program. For a more extensive exploration of determinants of HIV infection, adherence, and persistence, these should be collected in the future.

In summary, our findings provide important data on HIV incidence and adherence among KPs across a large number of health care settings and participants, supporting the inclusion of PrEP in the range of HIV preventive interventions. Suboptimal consumption or PrEP discontinuation contributes to reduced efficacy in real-world settings. It is essential to step up efforts to increase adherence and guarantee equitable access to PrEP for everyone who can benefit from it. These results are significant for public health, especially given the ongoing global PrEP scale-up to achieve zero HIV transmission by 2030.

## Funding

The Indonesian PrEP pilot program was funded by the Ministry of Health of Indonesia and the Joint United Nations Program on HIV/AIDS, Indonesia. The Indonesian PrEP pilot program was sponsored by the Global Fund to Fight AIDS, Tuberculosis and Malaria, USAID-PEPFAR, and the Government of Australia's Department of Foreign Affairs and Trade. The Indonesia PrEP Pilot Program Study Group acknowledges the support of the Kerti Praja Foundation, Padjajaran University, and the National MSM-TG Network. This work was also financially supported by the Program Hibah Dosen Tidak Tetap Peneliti 2024, managed by the Directorate for Multidisciplinary Science and Technology Implementation, Institut Teknologi Bandung, the DAPT EQUITY Program, Indonesia Endowment Fund for Education, the Ministry of Finance of Indonesia.

## Ethical approval statement

The research protocol was reviewed and all versions of the study documents (information sheet, consent form, and questionnaires) were approved by the designated research ethics committee (Padjajaran University Research Ethics Committee IRB no. 655/UN6. KEP/EC/2024) to implement, monitor, and evaluate the program at participating health care facilities. All the participants provided written informed consent.

## Author contributions

RW, MH, MP, TP, FR, DA, NA, EL, and BP conceived, designed, and managed data collection for the Indonesian PrEP pilot program. NH, MH, and RW contributed to the conception of the analyses. NH, AP, ZZ, KA, MH, and RW contributed to data interpretation. NH conducted all the analyses and drafted the manuscript. All the authors participated in the revision and final approval of the manuscript.

## Declarations of competing interest

The authors have no competing interests to declare.

## Appendix Supplementary materials

Image, application 1

Image, application 2

Image, application 3
