# Supplementary material for: HIV incidence and adherence after pre-exposure prophylaxis initiation in key populations in Indonesia: Findings from a real-world pilot program 2021-2023
Source: IJID Reg. 2025 Jan 19;14:100573. doi: 10.1016/j.ijregi.2025.100573 (PMC11848754; doi:10.1016/j.ijregi.2025.100573)
Supplement: Supplementary file 1 [file mmc1.docx]

**HIV Pre-exposure prophylaxis implementation for people at high risk of HIV infection in a resource-limited setting: protocol for a real-world pilot program in Indonesia 2021 – 2023**

**Rudi Wisaksana ^1^, Miasari Handayani ^1^, Mawar N. Pohan ^1^, Tarinanda A. Putri ^1^, Fani F. Rakhmat ^1^, Dwi S. Anggiani ^2^, Nurhalina Afriana ^2^, Endang Lukitosari ^2^, Bagus R. Prabowo ^3^ and Nadia Hanum ^4,5^***

^1^ Research Center for Care and Control of Infectious Disease, Padjajaran University, Indonesia

^2^ HIV and STI Working Group of the Directorate of Disease Prevention and Control, Ministry of Health, Indonesia

^3^ The Joint United Nations Programme on HIV/AIDS (UNAIDS), Indonesia

^4^ Pharmacology and Clinical Pharmacy – School of Pharmacy, Bandung Institute of Technology, Indonesia

^5^ Bioscience and Biotechnology Research Center, Bandung Institute of Technology, Indonesia

1. **Introduction**

High-quality evidence supporting the feasibility of PrEP (i.e., the efficacy, adherence retention, and risk compensation) implementation in Indonesia is lacking [1]. A PrEP pilot program, especially among high-risk KPs in Indonesia, therefore is needed to provide a new HIV prevention option, reduce new HIV infections, and provide the necessary evidence to inform the PrEP guidelines, including the costs of delivering PrEP within health service facilities. The specific objectives of this pilot study are to collect comprehensive, empirical data to determine effective ways to implement unrestricted HIV PrEP in Indonesia, including PrEP uptake and retention, the HIV prevention cascade, the real-life effectiveness of daily or event-driven PrEP use, safety and tolerability of PrEP, adherence to PrEP and the potential effects of PrEP use on sexual behaviours and incidence of sexually transmitted infections (STIs).

1. **Materials and Methods**
   1. *Overview of the study design*

The Indonesia PrEP Pilot Program is a longitudinal, multisite, real-world study of oral daily (D) or event-driven (ED) tenofovir disoproxil fumarate and emtricitabine (TDF/FTC) as PrEP among HIV-negative KPs at high risk of HIV infection in Indonesia. It was rolled out between December 2021 and December 2023, conducted by the Indonesia Ministry of Health of Indonesia (MoH), supported by the Global Fund to Fight AIDS, Tuberculosis and Malaria, USAID-PEPFAR and the Government of Australia’s Department of Foreign Affairs and Trade (DFAT) [2,3].

- - 1. Population and setting

KPs invited to participate in the study are MSM, FSW, ‘Waria’ (shemale/transgender women), PWID, and the serodiscordant partners of people with HIV. To be able to access the PrEP program, individuals should:

1. be HIV-negative aged 17 years and older
2. be at substantial risk of HIV transmission by reporting at least one of the following in the past three months: more than one sexual partner, inconsistent condom use, condomless anal sex, STIs history, ever used PrEP or post-exposure prophylaxis (PEP), and having an HIV positive partner who is not using antiretrovirals (ARVs), or using ARVs irregularly in the past six months, or without known viral loads (VLs), or with unsuppressed VLs (>1000 copies/mL) after six months of treatment, or planning to have children with unsuppressed VLs.
3. have no indications of having acute HIV infection and no contraindications to the regimen.

In this pilot program, 60 healthcare facilities spread across 21 districts in 10 provinces in Indonesia are included (**Table 1**). The selection of provinces is based on the consideration that the provinces have a fairly high HIV prevalence, which are DKI Jakarta, Bali, West Java, Central Java, DI Yogyakarta, East Java, South Sulawesi, East Kalimantan and Riau. Healthcare facilities (hospitals, clinics or public health centres / ’puskesmas’) included in the pilot program are those designated by the MoH as per recommendation from the Provincial Health Offices (PHOs) and the District Health Offices (DHOs). Recommendations are given if health facilities have been assessed to be ready to deliver PrEP services, as the health facilities: have the HIV Care, Support and Treatment (CST) and STIs treatment services, are frequently accessed or have a high number of coverage of KPs, have link and network with HIV communities, have received training in PrEP management (for the health workers), and are willing to be involved in the full implementation of the pilot program. The selected health facilities receive support from the MoH in the form of training, logistics including media for communication, information and education (CIE) and laboratory equipment required for HIV, STIs and HBsAg tests, operational supports, and regular assistance and monitoring from the MoH, PHOs, DHOs, and the Ministry of Health’s HIV Expert Panel.

**Table 1. List of Healthcare Facilities Providing HIV PrEP Services**

| **Provinces** | **Districts** | **Healthcare facilities** |
| --- | --- | --- |
| Jakarta | Jakarta Pusat | Puskesmas Kec. Sawah Besar |
|  |  | Puskesmas Kec. Senen |
|  | Jakarta Timur | Puskesmas Kec. Cakung |
|  |  | Puskesmas Kec. Cipayung |
|  | Jakarta Barat | Puskesmas Kec. Taman Sari |
|  |  | Puskesmas Kec. Tambora |
|  | Jakarta Selatan | Puskesmas Kec. Kebayoran Baru |
|  |  | Puskesmas Kec. Mampang Prapatan |
|  |  | Puskesmas Kec. Tebet |
|  | Jakarta Utara | Puskesmas Kec. KOJA |
|  |  | Puskesmas Kec. Pademangan |
|  |  | Puskesmas Kec. Penjaringan |
| Jawa Barat | Kota Bandung | Klinik Mawar PKBI Kota Bandung |
|  |  | Puskesmas UPT Garuda |
|  |  | RSU Dr. Hasan Sadikin |
|  | Kab. Bogor | RSU Ciawi |
|  |  | RSUD Leuwiliang Bogor |
|  |  | RSU Cibinong |
|  | Kota Bogor | Puskesmas Bogor Timur |
|  |  | Puskesmas Bogor Tengah |
|  | Kota Bekasi | RSUD Kota Bekasi |
|  |  | Puskesmas Pengasinan |
|  |  | Puskesmas Karang Kitri |
|  |  | Puskesmas Perumnas II |
|  | Kota Depok | RSUD Kota Depok |
|  |  | RS Sentra Medika Cisalak |
| Banten | Kota Tangerang | Puskesmas Tanah Tinggi |
|  |  | Puskesmas Kunciran Baru |
|  |  | Puskesmas Karawaci Baru |
|  |  | Puskesmas Cibodasari |
| Jawa Tengah | Kota Semarang | Puskesmas Halmahera |
|  |  | Puskesmas Poncol |
|  |  | Puskesmas Kedungmundu |
|  |  | Puskesmas Lebdosari |
| D.I Yogyakarta | Kota Yogyakarta | Puskesmas Tegalrejo |
|  |  | Puskesmas Umbulharjo I |
|  |  | Puskesmas Gedong Tengen |
| Jawa Timur | Kota Surabaya | Puskesmas Sememi |
|  |  | Puskesmas Kedungdoro |
|  |  | Puskesmas Perak Timur |
|  |  | Puskesmas Putat Jaya |
|  | Kab. Sidoarjo | RSU Sidoarjo |
|  |  | Puskesmas Sedati |
|  |  | Puskesmas Balongbendo |
|  |  | Puskesmas Gedangan |
| Bali | Kab. Badung | Puskesmas Kuta I |
|  |  | Puskesmas Kuta II |
|  |  | Puskesmas Kuta Selatan |
|  | Kota Denpasar | Puskesmas Denpasar Utara I |
|  |  | Klinik Utama WM Medika Yayasan Kerti Praja (YKP) |
| Sulawesi Selatan | Kota Makassar | Puskesmas Jumpandang Baru |
|  |  | Puskesmas Makkasau |
|  |  | Puskesmas Andalas |
| Kalimantan Timur | Kota Balikpapan | RSUD. Dr. Kanujoso Djatiwibowo |
|  |  | Puskesmas Prapatan |
|  |  | RS Tk. III Dr. R. Hardjanto |
|  | Kota Samarinda | Puskesmas Palaran |
|  |  | Puskesmas Temindung |
| Kep. Riau | Kota Batam | Puskesmas Lubuk Baja |
|  |  | RS Budi Kemuliaan Batam |

- - 1. Sample size

As this is a pilot and feasibility study, the sample size is not determined by power calculations. The aim is to get 26,000 people on PrEP by the end of 2023 to assess feasibility across a diverse range of participants including different KPs, regions and ages. The target of 26,000 people is based on the estimated size of KPs and the number of health facilities that can provide CST services in the 10 provinces included. In 2022, the estimated size of KPs was 227,600 for FSW, 761,000 for MSM, 27,100 for PWIDS, and 34,000 for transgender people [4].

- - 1. Ethics approval of the research

The research protocol and all versions of the study documents (information sheet, consent form, and questionnaires) were approved by the designated research ethics committee (Padjajaran University Research Ethics Committee). Based on this, the study subsequently received permission for implementation study at the participating healthcare facilities.

- 1. *Implementation of the PrEP program*
     1. PrEP Program Model

Table 2 depicts the PrEP model delivery in Indonesia and its flow. Briefly, PrEP services are provided by healthcare facilities but with the involvement of the HIV community, which is very important to achieve the goals and targets of PrEP services (health facilities and community-led-based). Community-led AIDS responses are vital for addressing stigma and discrimination; providing treatment education and adherence support and prevention interventions; supporting differentiated service delivery; and reaching all people who need those services [5 – 7]. Indonesia has already completed a full cycle of community-led monitoring, but meaningful engagement of young people from key populations in decision-making platforms remains limited [4,8].

**Table 2. PrEP Implementation Model in Indonesia**

|  | Pre-enrollment | | Baseline | Follow-up | |
| --- | --- | --- | --- | --- | --- |
|  | **Promotion and education** | **HIV test and counselling** | **First visit** | **First follow-up** | **Next follow-up** |
| When | recruitment stage | Day 0 | Day 1 (starting PrEP) | Month 1 | Month 3 and next routine visits every 3 months or incidental |
| Where | HIV communities, CST services, or mobile services | HIV communities, CST services, mobile services | CST services, mobile services | | |
| Who  (deliver the service) | Community outreach workers, peer educators, or HIV counselors | Community outreach workers, peer educators, HIV counselors, health workers (doctors, nurses, lab staff) | Health workers (doctors, nurses, lab staff, pharmacists, reporting and recording (RR) officers) | Health workers (doctors, nurses, lab staff, pharmacists, RR officers), HIV counselors, peer educators, and community outreach workers | |
| What  (service package) | Information, Education and Communication (IEC) package, risk behaviour screening | HIV test, medical screening | Consent form, counseling and education, PrEP medication, referral tests (optional) | PrEP medication, monitoring for side effects, adherence, referral tests (STIs, Hep B, Hep C, creatinine | |
| How  (service delivery) | Campaigns on social media and through HIV communities, in-person outreach | Available at designated healthcare facilities and included in the government program | | | |
| ** this includes acute HIV examination and counseling for PrEP education and behavior screening* | | | | | |

- - 1. Promotion and recruitment of participants

In this stage, peer educators and outreach workers in the community carry out promotional and educational activities to KPs by providing CIE packages through mobile (via phone / social media) or in-person reach out or group meetings (Table 1). Interested individuals will be directed to come to the designated healthcare facilities which provide PrEP to undergo PrEP screening and HIV tests. At the facilities level, health workers will continue promoting and educating individuals referred by community workers. Voluntary Counselling and Testing (VCT) clients and serodiscordant couples who access HIV services at health facilities are also targets for recruitment.

- - 1. Screening
- Risk behavior screening

This screening is done to evaluate whether a prospective user is at elevated risk of HIV through an online questionnaire regarding risky behavior. Prospective users can complete the questionnaire independently through a mobile phone or computer (they will receive a link to a webpage), with peer educators/outreach workers in the community, or with HIV counsellors in health facilities. If the online questionnaire cannot be accessed, the questionnaire can also be accessed in paper form. The questionnaire is in Indonesian language / ‘Bahasa’. Those who report at least one risky behavior are classified as eligible to start PrEP and will then undergo medical screening. Individuals who are not considered to have a greater risk of HIV will be advised to use other prevention methods such as condoms.

- Medical screening

This screening is carried out at CST services and includes screening of HIV, STIs, Hepatitis B and C, and creatinine for those aged under 30 years who have a history of comorbidities such as hypertension and diabetes. Those with HIV-positive results will be referred to HIV treatment, those known to have an STI or positive for Hepatitis B and C will also be referred for treatment (negative Hepatitis B is a prerequisite for starting PrEP for prospective event-driven PrEP users), and creatinine clearance result of <60 ml/minute means a person cannot start/continue PrEP and will be referred to a specialist in internal medicine.

- 1. *Study enrollment and follow-up*

Prior to PrEP initiation, prospective PrEP users sign an informed consent and will be counseled by health workers regarding the program’s procedure and detailed instructions for PrEP use. Those who initiate PrEP can choose to take PrEP on a daily basis (D-PrEP) or according to an event-driven regimen before and after anal sex (ED-PrEP), however the decision regarding the accepted regimen depends on the considerations of the health worker/counselor. This is due to the need for consideration between the choice of regimen and the behavioral patterns of prospective users, especially those who choose ED-PrEP. Potential users who do not fit the criteria for PrEP-ED users will be recommended to use a daily PrEP regimen.

Recruitment started on 1 December 2021 and follow-up ended on 31 December 2023. Participants completed baseline (screening) and follow-up visits at month one and then every three months (months 3, 6, 9, 12 and so on). For each clinic visit, adverse events and concomitant medication are documented and HIV/STI testing, alongside other clinical evaluations based on indications. Participants are asked to report the past three months’ sexual behaviours (number of sexual partners, frequency sex, condomless anal sex), side effects and adherence. Participants are required to return the leftover TDF/FTC to the clinic in exchange for a refill to cover their needs until the next clinic visit. Besides the three monthly follow-up visits, non-routine visits can also be made if there are side effects from taking medication, risk reduction and adherence counselling, and for PrEP-ED users to take medication. In its implementation, providing PrEP-ED requires a strong counsellor role, especially in determining whether they meet the criteria for PrEP-ED users and how to strengthen user compliance. Counsellors reserve the right to recommend users switch regimens according to their sexual behavior patterns and PrEP consumption.

If a participant is found to have seroconverted to HIV, his participation in the study will be terminated immediately. At the final visit, study staff collect unused pills, conduct resistance and viral load testing, and provide referrals to HIV services. PrEP use during follow-up can be terminated for other reasons including having problems with compliance, feeling that they are no longer at risk due to the changes in behaviours or lifestyle, experiencing side effects that cannot be overcome and interfere with daily activities, and blood test results show that there are adverse drug effects in the body.

- 1. *PrEP Mobile Application for PrEP Users*

After being registered as a PrEP user, clients will receive a national registration number which can be used to access PrEP services in this pilot program. This registration number is also used as the username on the PrEP mobile application. This PrEP mobile application can be downloaded by PrEP users from the Play Store, but is currently still available for Android (**Figure 1**).

This PrEP mobile application functions:

1. To provide information about the conditions for taking PrEP medication.
2. As a calendar to mark sexual activity and PrEP medication taking patterns, as well as to book visits to the clinic. The visit booking menu on the PrEP mobile application is connected to the reporting recording application at the targeted PrEP provider's health service, so that health workers will be able to find out the client's visit schedule automatically after the user makes a booking.
3. As a reminder of the clinic visit schedule.


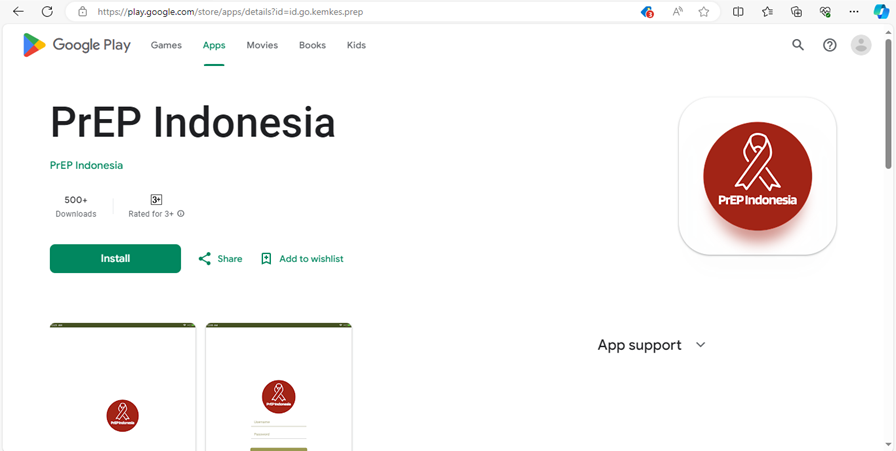


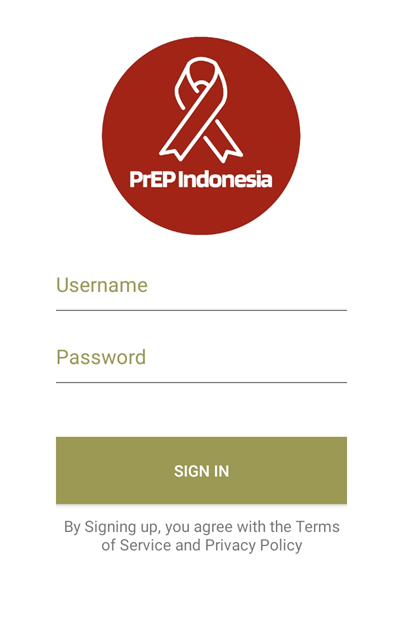


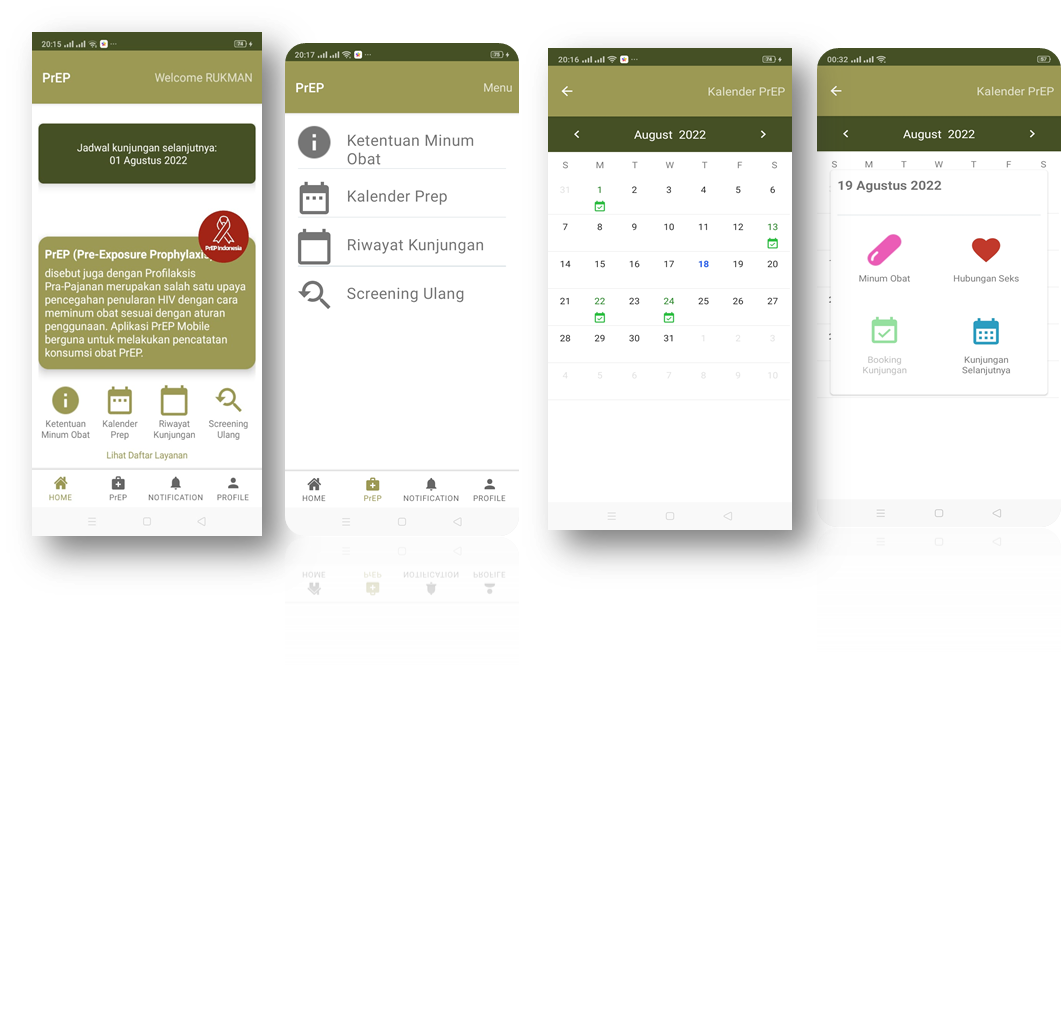


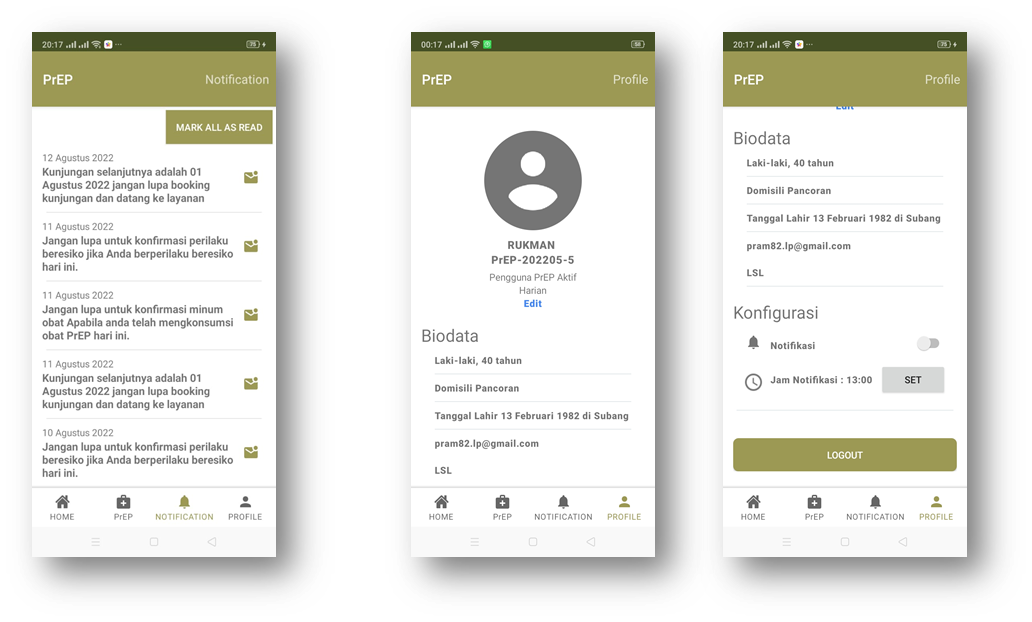


**Figure 1. Home Page and Application Menu in the Mobile PrEP App**

- 1. *HIV and STIs testing*

HIV and STIs tests were done following the national guideline. Rectal gonorrhea or chlamydia infection was diagnosed by NAAT-positive rectal swab that was either provider-collected or self-collected, depending on participant preference. Venipuncture blood specimens were tested for antibody to Treponema pallidum (T. pallidum) byrapid plasma reagin (RPR) reactivity and titer and T. pallidum antibody rapid test. Persons who also tested T. pallidum antibody positive and with RPR ≥1:8 were considered seropositive for syphilis. Participants with positive STI results were provided oral or injection treatment following local clinical standards.

- 1. *Study termination*

If a participant seroconverted to HIV, his participation in the study would be terminated immediately. At the final visit, study staff collected unused pills and provided referrals to HIV services. Other reasons for termination included having problems with compliance, feeling that they were no longer at risk due to the changes in behaviours or lifestyle, experiencing side effects that cannot be overcome and interfere with daily activities, blood test results showed that there are adverse drug effects in the body, and lost to follow-up (no study visits for three consecutive follow-up).

- 1. *Monitoring and Evaluation*

The Monitoring and Evaluation (ME) process is done to monitor the PrEP coverage among target KPs, to identify clinical or structural areas for service improvement, and to evaluate the delivery of the program and its impact. Implementation of the PrEP program follows a cascade that is almost the same as the HIV treatment [31] with primary and secondary endpoints as below.

- - 1. Key indicators of PrEP program coverage

To assess the applicability and feasibility of a national PrEP roll-out, the key endpoints are:

1. PrEP eligibility, acceptability, and initiation
2. PrEP continuation and retention
3. The rate of side effects or adverse events related to PrEP use
4. HIV Incidence

The secondary endpoints are:

1. Adherence to PrEP, which will be assessed through self-report to the clinician or HIV counsellor, dispensing and return records, the proportion of participants who switch regimens and their reasons, and drug level assessment
2. Dynamics of PrEP use and patterns in follow-up, including switching between regimens, discontinuing and restarting PrEP and the rate of adverse events related to discontinuation of PrEP or switching PrEP regimens
3. Factors associated with HIV Incidence
4. The potential effects of PrEP use on sexual behaviours and the incidence of STIs
   - 1. Data management, safety and monitoring

All study participants are assigned a unique code to enable data linkage throughout follow-up. All data (questionnaires and clinical data) are entered into an electronic management system developed by the Research Center for Care and Control of Infectious Disease (RC3ID) of Padjajaran University by the recording and reporting staff at the healthcare facilities and will be assisted by research team members who are trained in data entry. Data collection and study conduct are monitored through monthly meetings with the research team, representatives from the community, research nurses and project pharmacists to ensure protocols are implemented consistently. Data collected as part of this study will be treated confidentially and stored securely at the RC3ID. All study staff are trained in confidentiality, have signed confidentiality agreements, and have been trained in ethical human subject research practices to minimize participant risk.

- - 1. Data analyses

Quantitative data will be imported into the STATA Statistical Software (StataCorp, College Station, Texas, USA) for analysis. Demographic information obtained from questionnaires will be analysed using descriptive statistics. For PrEP uptake, we will perform appropriate statistical tests to examine group differences in proportions over the course of the study. Statistical analysis of the risk of HIV seroconversion, sexual behavior and adherence to the study will be conducted using longitudinal models with time-varying exposures. All data submitted to the study will be collated before analysis, and the effects of the missing data will be investigated.

Data collection activities ended in January 2024, while data cleaning and analyses have begun since February 2024. Results will be disseminated through a national report and policy brief, peer-reviewed journals, and presentations at local and international conferences to national and global policymakers, community and participants. We will also create easy-to-read infographics to share via social media and our research group website(s).

1. **Discussion**

This pilot program is the first oral TDF/FTC combination as a PrEP study in Indonesia, which will provide primary data on PrEP implementation, including the cascade of HIV Prevention, real-world effectiveness, safety, and adherence. The findings from this study will be vital for promoting the integration of PrEP within the portfolio of HIV prevention interventions and developing guidance on unrestricted PrEP implementation in Indonesia. Furthermore, results from this study will inform global health policy and provide reassuring evidence of PrEP effectiveness among diverse populations in resource-limited settings. The limitations of this study include, first, as a pilot project, this study has a follow-up restricted to a maximum of two years, so there is limited potential to detect long-term trends and adherence to PrEP. Despite this, frequent detailed data collection, particularly on behavioural practices and STI testing and diagnosis, and procedures to maintain high rates of follow-up will ensure that a high-quality and complete data set is available for analysis. This is a one-arm study and there is no comparison group, but as this pilot program is a feasibility and implementation study, this group is not necessary.

1. **Conclusions**

In conclusion, the Indonesia PrEP Pilot Program is designed to evaluate the feasibility of an Indonesian model of prescribing oral PrEP to individuals at high risk of HIV transmission. Given the planned extensive data collection and analysis, this study will provide crucial information on PrEP implementation in Indonesia and the impacts of PrEP use on the wider community. It will also play a major role in developing and assessing the Indonesian National PrEP Guidelines, resulting in optimal access to PrEP for those who need it most.

**References**

1. Cempaka R, Wardhani B, Sawitri AAS, Januraga PP, Bavinton B. (2020). PrEP Use Awareness and Interest Cascade among MSM and Transgender Women Living in Bali, Indonesia. Trop Med Infect Dis. 2020;5(4):158. Published 2020 Oct 10. doi:10.3390/tropicalmed5040158
2. Indonesian Ministry of Health - Directorate General Disease Prevention and Control. (2021). Technical Instructions for the Management of the Pre-Exposure Prophylaxis Pilot Program (PrEP) for People at High Risk of HIV Infection in Indonesia.— Jakarta : Indonesian Ministry of Health.
3. UNAIDS. Successfully expanding the rollout of PrEP in Indonesia, 2022. Available from: <https://www.unaids.org/en/resources/presscentre/featurestories/2022/september/20220901_prep-indonesia#:~:text=PrEP%20was%20rolled%20out%20in,who%20have%20sex%20with%20men>
4. UNAIDS. UNAIDS DATA 2023, 2023. Available from: <https://www.unaids.org/en/resources/documents/2023/2023_unaids_data>
5. UNAIDS. Community-led AIDS Responses, 2022. Available from: <https://www.unaids.org/sites/default/files/media_asset/community-led-aids-responses_en.pdf>
6. Anam FR, Nkosi S, Sebayang M, Jokonya M, Dunaway K, El Alaoui T. (2023). Let us lead: community leadership in the AIDS response is its fundamental pillar for success. J Int AIDS Soc. 2023;26(12):e26196. doi:10.1002/jia2.26196
7. Ayala G, Sprague L, van der Merwe LL, et al. (2021). Peer- and community-led responses to HIV: A scoping review. PLoS One. 2021;16(12):e0260555. Published 2021 Dec 1. doi:10.1371/journal.pone.0260555
8. Bangkok: Youth LEAD. Engagement of youth in the Country Coordinating Mechanism and Global Fund processes in the Asia Pacific, 2022. Available from: <https://www.youthleadap.org/news-updates/meaningful-engagement-youth-ccm>
