# Supplementary material for: HIV incidence and adherence after pre-exposure prophylaxis initiation in key populations in Indonesia: Findings from a real-world pilot program 2021-2023
Source: IJID Reg. 2025 Jan 19;14:100573. doi: 10.1016/j.ijregi.2025.100573 (PMC11848754; doi:10.1016/j.ijregi.2025.100573)
Supplement: Supplementary file 2 [file mmc2.docx]

**Supplemental File 2**

**1,115 not eligible based on risk assessment**

**7,345 did not enter PrEP care**

**Fig. 1: The Indonesia PrEP Pilot Program – Study Profile (2021 – 2023)**


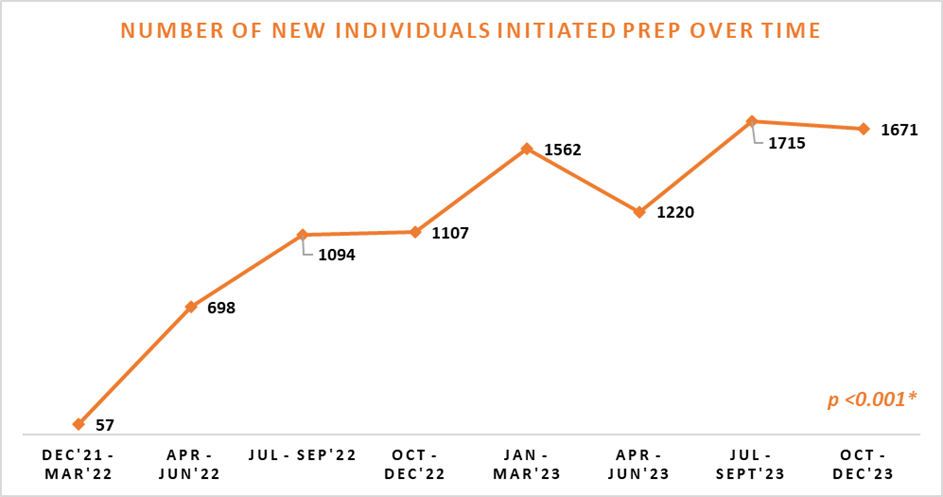


****p-value for linear trend***

**Fig. 2: Number of new participants initiated PrEP in the Indonesia PrEP Pilot Program (2021 – 2023)**

**Table 1: HIV Incidence and associated predictors (using mixed-effects Poisson regression models)***

|  | HIV Incidence | PYs | Incidence rate per 100 PYs (95% CIs) | Adjusted IRR*  (95% CI) | p-values |
| --- | --- | --- | --- | --- | --- |
| Age (as continuous variable)  Age category  <25 years  ≥25 years | 10  21 | 572.93  2209.81 | 1.75 (0.8 – 3.2)  0.9 (0.6 – 1.45) | 7,582 obs  0.94 (0.87 – 1.01)  Ref  0.48 (0.22 – 1.03) | 0.06 |
| Regimen  Daily  Event – Driven | 20  11 | 2112.36  651.13 | 0.95 (0.58 – 1.46)  1.69 (0.80 – 3.02) | 7,136 obs  Ref  0.56 (0.26–1.21) | 0.138 |
| Region  Java – Bali  Outside Java – Bali | 28  3 | 2715.51  80.22 | 0.11 (0.02 – 0.32)  3.75 (0.7 – 10.9) | 7,582 obs  Ref  5.56 (1.68 – 18.38) | **0.005** |
| Multiple sex partners  No  Yes | 11  20 | 1699.23  1096.50 | 0.6 (0.30 – 1.15)  1.8 (1.11 – 2.81) | 7,582 obs  Ref  1.27 (0.59 – 2.75) | 0.530 |
| Condom use at last sex  Yes  No | 7  24 | 234.54  2559.89 | 2.98 (1.19 – 6.13)  0.98 (0.6 – 1.39 | 7,426 obs  Ref  0.55 (0.22 – 1.34) | 0.188 |
| Consistent condom use  Yes  No | 6  25 | 957.95  1837.78 | 0.6 (0.23 – 1.36)  1.36 (0.88 – 2.01) | 7,624 obs  Ref  1.17 (0.45 – 3.06) | 0.751 |
| STI diagnoses  No  Yes | 30  1 | 2638.78  156.94 | 1.11 (0.77 – 1.62)  0.6 (0.02 – 3.54) | 7,136 obs  Ref  0.79 (0.10 – 5.82) | 0.820 |
| Adherence status  Poorer or very low  Adequate | 31  0 | 883.77  394.27 | 7.8 (5.34 – 11.16)   1. (0 – 0.4) | (omitted due to collinearity) |  |
| ** Determinants were analysed among MSM only due to very low number of incidents among other KPs*  *** aIRR adjusted for age (as a continuous variable) and adherence status, aIRR for age was adjusted for adherence status only* | | | | | |

**Table 2: STI Incidence among key populations**

|  | Person-Years (PYs) | No. of STI Incidence | Incidence rate per 100 PYs  (95% CIs) |
| --- | --- | --- | --- |
| Total | 2474.51 | 715 | 28.89 (27.11 – 30.72) |
| Key populations | | | |
| MSM | 1870.21 | 633 | 35.45 (33.28 – 37.67) |
| FSW | 235.74 | 20 | 8.47 (5.25 – 12.78) |
| PWID | 2.50 | 0 | 0 (0 – 70.76) |
| TGW | 105.37 | 16 | 15.24 (8.97 – 23.56) |
| sero-discordant partners | 260.69 | 46 | 17.62 (13.20 – 22.80) |


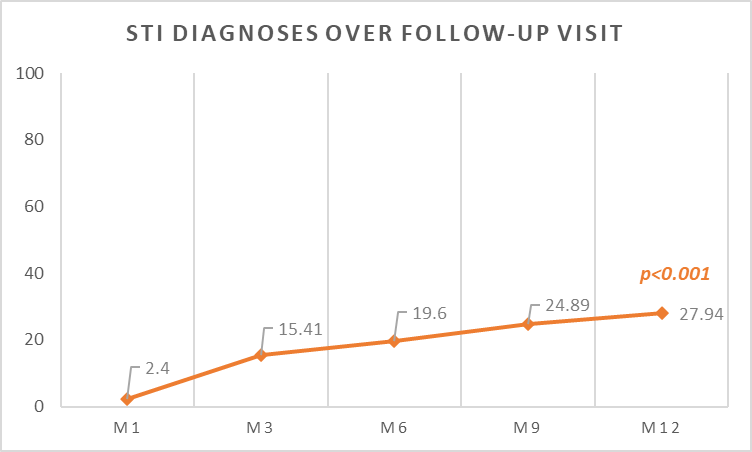


****p-value from GEE-logistic model***

**Fig. 3: STI diagnoses over follow-up period**

**Table 3. HIV Incidence in Bali and Jakarta among MSM and TGW compared to previous cohort study among MSM and TGW in Jakarta and Bali between 2017 and 2020**

|  | Indonesia PrEP Pilot Program | Non-PrEP users cohort |
| --- | --- | --- |
| Area | Jakarta and Bali | Jakarta and Bali |
| Key populations | MSM and TGW | MSM and TGW |
| n | 2,291  (1,418 in Jakarta and 873 in Bali | 2,341  1,807 in Jakarta and 534 in Bali |
| HIV Incidence rates  Jakarta (95% CI)  Bali (95% CI) | 9.39/100 PYs (7.78 – 11.17)  7.24/100 PYs (5.73 – 9.13) | 1.69/100 PYs (1.05 – 2.56)  0.65 (0.07 – 2.36) |
